# First selective direct mono-arylation of piperidines using ruthenium-catalyzed C–H activation

**DOI:** 10.1007/s00706-013-0947-1

**Published:** 2013-04-03

**Authors:** Maria C. Schwarz, Navid Dastbaravardeh, Karl Kirchner, Michael Schnürch, Marko D. Mihovilovic

**Affiliations:** Institute of Applied Synthetic Chemistry, Vienna University of Technology, Getreidemarkt 9/163-OC, 1060 Vienna, Austria

**Keywords:** Catalysis, Heterocycles, C–H activation, Metal carbonyls, Arylation, Detrifluoromethylation

## Abstract

**Abstract:**

A Ru-catalyzed mono-arylation in α-position of saturated cyclic amines is reported employing a C–H activation protocol. Substitution of the pyridine directing group with a bulky group, e.g., trifluoromethyl in the 3-position, proved to be crucial to avoid bis-arylation. This highly selective transformation can be performed with different amines and arylboronate esters. Additionally, the directing group can be cleaved, taking advantage of an unprecedented detrifluoromethylation reaction.

**Graphical Abstract:**

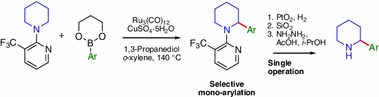

## Introduction

Transition-metal-catalyzed C–H activation has become an important tool for organic chemists in recent years [1–8]. Activation of sp^2^ C–H groups is already well established [9–17], and though transition-metal-catalyzed sp^3^ C–H activation is more difficult, there are also various recently reported examples [18–31]. This approach offers an appealing method towards more efficient synthetic pathways with fewer steps, since pre-activation of carbons with functional groups can be avoided.

α-Substituted saturated N-heterocycles can be found in natural products such as alkaloids, as well as in drug compounds [32, 33]. Hence, utilization of the sp^3^ C–H bond for C–C bond-forming reactions in α-position to the nitrogen is of special interest, since it provides an efficient pathway towards a valuable building block that is tedious to obtain via other methods [34].

Due to the importance of α-arylated cyclic amines, several groups have undertaken efforts to develop direct arylation methods towards these compounds. Sames and coworkers reported the first direct α-arylation via C–H activation of saturated cyclic amines, primarily pyrrolidines, using a ruthenium catalyst and arylboronate esters as coupling partner (Scheme 1, upper part) [35]. A cyclic imine was used as a directing group, and the presence of a ketone proved to be essential for this reaction. In 2010, the group of Maes published a ruthenium-catalyzed, pyridine-directed C–H activation of piperidine derivatives in the presence of an alcohol, again involving arylboronate esters as coupling partners (Scheme 1, lower part) [36].

**Figure:**
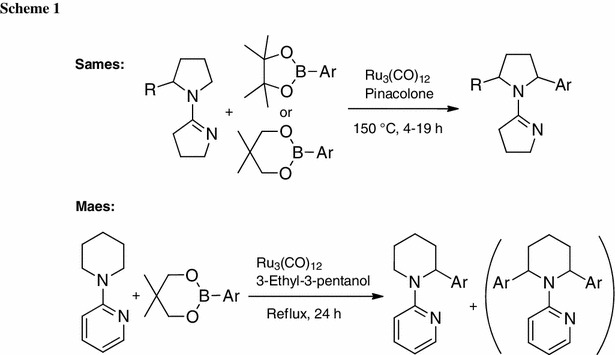

Both of these methods have one notable limitation; i.e., no selective mono-arylation could be achieved. In one case the second α-position was blocked to avoid bis-arylation (Sames’ protocol); in the other case a mixture of mono- and bis-arylated products was obtained (Maes’ protocol). Bis-arylation not only decreases the yield of the mono-arylated compound but also leads to difficult separation due to similar properties of mono- and bis-arylated compounds. The best yield for selective mono-arylation reported to date was published by Maes with 49 % [arylation of (pyridin-2-yl)piperidine with 3-(trifluoromethyl)phenyl boronic acid ester] [36]. Herein, we report on selective mono-arylation of saturated cyclic amines achieved using a specially designed directing group.

## Results and discussion

Bis-arylation occurs due to a low energy barrier for rotation of the directing group around the C–N bond as observed for pyridine or the cyclic imine. Free rotation therefore allows insertion of the catalyst into both C–H bonds after complexation of the catalyst to the nitrogen of pyridine (Scheme 2). For this reason, it was envisioned to install a bulky group in 3-position of the pyridine directing group to hamper this free rotation, subsequently avoiding a second arylation step. The trifluoromethyl group was chosen for several reasons: (i) similar directing groups have already been successfully applied in direct arylation reactions [37–39]; (ii) it gave better results as compared with the simpler CH_3_ group (typically ~10 % better conversion); (iii) only mono-arylated product was obtained in the initial screening; and (iv) it is easy to install in good yields.

**Figure:**
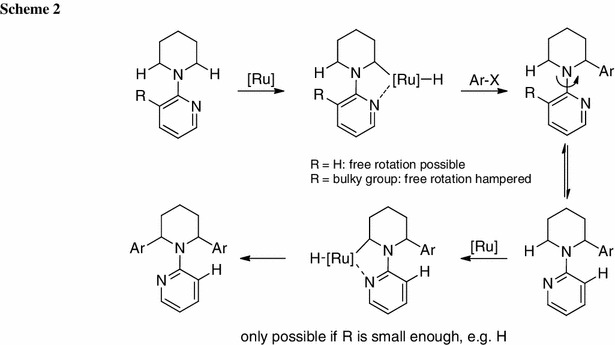

In most cases the directing group was installed by simple nucleophilic aromatic substitution (Table 1). Therefore, 2-chloro-3-(triflouromethyl)pyridine (**1**) was reacted with saturated amine **2**, adding K_2_CO_3_ and using acetonitrile as a solvent. Except **3b** (Table 1, entry 2) and **3k** (Table 1, entry 11), all products could be obtained in excellent yields. The rather low yield of **3b** is probably due to steric hindrance of the methyl group adjacent to the piperidine nitrogen. Though **3k** showed conversion of 74 %, only 37 % of pure product could be isolated, which can be attributed to significant volatility of the product leading to losses upon solvent evaporation.

**Table 1:**
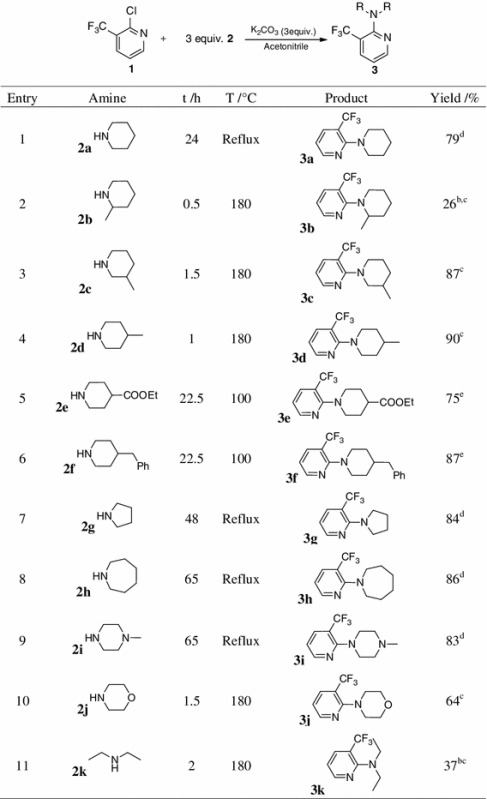
Synthesis of substrates for direct arylation

^a^Reaction conditions: **1** (1 equiv.), saturated amine **2** (2 equiv.), K_2_CO_3_ (2 equiv.), acetonitrile

^b^3 equiv. saturated amine

^c^Microwave heating

^d^Round-bottom flask, reflux conditions

^e^Closed vial, heating block

Having a series of starting materials in hand, the direct arylation reaction was optimized. Initially, the C–H activation was performed with Ru_3_(CO)_12_ as catalyst and 1,3-propanediol derived boronic esters as the aryl donor, similar to previous literature examples [35, 36] and being easily prepared from the corresponding boronic acids [40]. As we observed also deborylation besides the desired arylation reaction, an excess of 4 equiv. of arylboronate ester was used. Maes reported that addition of an alcohol (e.g., 2,2-dimethylpropane-1,3-diol) was beneficial in his piperidine arylation protocol, since the alcohol should scavenge a diol–borane species formed after transmetallation [36]. Hence, we also tried addition of 2,2-dimethylpropane-1,3-diol. The reaction was performed in a reaction vial with a septum cap and an attached argon balloon to “release” hydrogen which should be formed according to the mechanism proposed by Maes. The reaction was carried out in *o*-xylene as solvent at 135 °C for 36 h. With those conditions (Table 2, entry 1) conversion of 53 % could be achieved. Changing the nature or amount of alcohol had no significant influence on the reaction outcome. In the Maes protocol, 1 equiv. of alcohol was used [36]. In our case it did not make any difference whether we used 0.5 or 1 equiv. of alcohol additive. Increasing the catalyst loading did not improve the yield either. Also, longer reaction times did not increase the yield (data not shown). It turned out that, after a significant amount of screened reaction conditions, the only modification that could increase the yield significantly was to add a metal salt as co-catalyst. Table 2 presents a selection of metal salts applied. Addition of PdCl_2_ (entries 2–4), FeCl_2_ (entries 5–7), FeCl_3_ (entries 8–10), CuCl_2_·2H_2_O (entries 11–13), and CuSO_4_·5H_2_O (entries 14–16) led to improved conversion, with 2 mol% CuSO_4_·5H_2_O giving the best gas chromatography (GC) yield of 70 %. It can only be speculated that the metal salt may help to keep the catalyst in the required oxidation state, but evidence for this is lacking. To investigate the role of CuSO_4_·5H_2_O and to exclude that CuSO_4_·5H_2_O alone could serve as a catalyst in this transformation, the reaction was carried out in the absence of Ru_3_(CO)_12_; as expected, no formation of the product could be observed.

**Table 2:** Optimization of the reaction conditions^a^

Entry	Co-catalyst	Mol%	Conversion/%^b^	GC yield/%^c^
1	–	–	53	39
2	PdCl_2_	1	53	43
3	PdCl_2_	3	70	54
4	PdCl_2_	5	65	49
5	FeCl_2_	1	72	54
6	FeCl_2_	2	75	61
7	FeCl_2_	5	75	63
8	FeCl_3_	1	73	58
9	FeCl_3_	2	77	58
10	FeCl_3_	5	71	49
11	CuCl_2_·2H_2_O	1	77	63
12	CuCl_2_·2H_2_O	2	72	61
13	CuCl_2_·2H_2_O	5	69	43
14	CuSO_4_·5H_2_O	1	74	59
15	CuSO_4_·5H_2_O	2	82	70
16	CuSO_4_·5H_2_O	5	87	69

^a^Reaction conditions: **3a** (0.5 mmol), **5a** (2 mmol), Ru_3_(CO)_12_ (7 mol%), co-catalyst, 2,2-dimethylpropane-1,3-diol (0.25 mmol), 0.5 cm^3^ *o*-xylene, 135 °C, stirred for 36 h under argon conditions in an open vial

^b^Conversion based on GC analysis with respect to **3a** and **4a** (dodecane as internal standard)

^c^Yield determined by GC analysis with respect to **4a** (dodecane as internal standard) using a calibration curve

A slight increase of the temperature to 140 °C still gave the same conversion, but the reaction time could be decreased to 24 h. Further increasing the temperature was not beneficial and lower conversions were obtained, which most likely can be attributed to catalyst decomposition. Since the kind of alcohol had no influence on the yield, 0.5 equiv. of 1,3-propanediol was used instead of 2,2-dimethylpropane-1,3-diol. This change was conducted as the 1,3-propanediol derived boronate was much easier to separate from the product in the workup process and the nature of the boronic ester had no significant influence on the substrate conversion. At this point it could be argued that the avoidance of bis-arylation is no advantage over existing protocols, since instead of having to separate mono-arylated from bis-arylated compound, the mono-arylated compound now has to be separated from starting material due to incomplete conversion. However, separation of substrate from mono-arylated product is very facile, whereas separation of mono- and bis-arylated products is very difficult.

With the optimized conditions in hand, we explored the scope of the reaction. For piperidine, different aryl groups were introduced and the results are shown in Table 3. The best result could be achieved for the unsubstituted phenyl group, yielding 60 % of the desired product **4a** (Table 3, entry 1). Also in the presence of electron-donating alkyl groups as substituents in *para*- or *meta*-position, yields in the range of 50 % were obtained for **4b**, **4c**, and **4j** (Table 3, entries 2, 3, 10). The presence of a substituent in *ortho*-position decreased the conversion dramatically, most likely due to steric hindrance (Table 3, entry 12, **4l**). Boronic acid esters bearing electron-withdrawing groups gave lower yields (Table 3, entries 4, 5, 7, 11). Nitrogen-containing electron-withdrawing groups such as nitro or cyano were not tolerated (Table 3, entries 8, 9). This may be due to complexation of the catalyst at these functional groups, making the catalyst unavailable for the desired transformation. The trend for electron-withdrawing substituents to give lower and electron-withdrawing coordinating substituents to give no conversion was already observed by us in the previously reported direct arylation of benzylamines [37–39]. The result for the *p*-methoxy substituent was surprising (Table 3, entry 6). Compound **4f** could be isolated with yield of only 16 %, because to some extent also the bis-arylated product was formed. This was the only case where bis-arylation was observed for piperidine, when employing a substituted pyridine directing group. To separate the two compounds, preparative thin-layer chromatography (TLC) had to be performed additionally after flash column chromatography. This accounts for the low yield and shows the isolation problems with unselective reactions. A pure fraction of bis-arylated product could actually not be obtained. The mono-arylated product **4f** seems to promote the second arylation step. This hypothesis was tested by subjecting isolated **4f** to the arylation conditions. Indeed, formation of the bis-arylated compound could be observed by GC–mass spectrometry (MS), albeit only in small amounts (~10 %).

**Table 3:** Scope of mono-arylations with different arylboronate esters **5** ^a^

Entry	Ar	Product	Yield/%^b^
1	C_6_H_5_	**4a**	60
2	4-Me-C_6_H_4_	**4b**	47
3	4-*t*-Bu-C_6_H_4_	**4c**	50
4	4-F-C_6_H_4_	**4d**	43
5	4-Cl-C_6_H_4_	**4e**	34
6	4-MeO-C_6_H_4_	**4f**	16^c^
7	4-CF_3_-C_6_H_4_	**4g**	40
8	4-CN-C_6_H_4_	**4h**	n.i.
9	4-NO_2_-C_6_H_4_	**4i**	n.i.
10	3-Me-C_6_H_4_	**4j**	49
11	3-Cl-C_6_H_4_	**4k**	39
12	2-Me-C_6_H_4_	**4l**	n.i.

^a^Reaction conditions: **3a** (0.5 mmol), **5** (2 mmol), Ru_3_(CO)_12_ (7 mol%), CuSO_4_·5H_2_O (2 mol%), 1,3-propanediol (0.25 mmol), 0.5 cm^3^ *o*-xylene, 140 °C, stirred for 24 h under argon conditions in an open vial

^b^Isolated yield after flash column chromatography; those examples with low or no conversion were not isolated (n.i.)

^c^Bis-arylated product was obtained in addition; additional purification by preparative TLC

The scope of the reaction was also investigated regarding different saturated amines (Table 4). In the case of 2-methyl-substituted piperidine **3b** (Table 4, entry 1), the conversion dropped, as expected, to only 36 % due to steric hindrance. When the arylation was conducted on 3-methyl-substituted piperidine **3c** (entry 2), four isomers could be observed on GC–MS, which indicated that, in addition to the expected 2,5-substituted compound, also a 2,3-substituted compound was synthesized, both in *cis* and *trans* conformation. Not surprisingly, the isomers could not be separated by flash column chromatography. Overall, the yield was 30 %. For 4-substituted piperidines (Table 4, entries 3–5), yields between 25 and 34 % were obtained. In all cases only one isomer was observed and isolated. ^1^H nuclear magnetic resonance (NMR) signal patterns and coupling constants revealed that the aryl group in **8** is *cis* to the methyl group in 4-position. The products of the other reactions were assigned as the *cis*-isomers by analogy. Compared with piperidine, pyrrolidine **3g** (Table 4, entry 6) showed higher conversion, but due to the different geometry of the ring, the trifluoromethyl group was not as effective in preventing bis-arylation (mono:bis = 1:0.35 on GC–MS). The mono-arylated compound **11** was obtained in 49 % yield, but the bis-arylated product could not be obtained in pure form. Azepane (**3h**), piperazine (**3i**), and morpholine (**3j**) substrates (Table 4, entries 7–9) gave only low conversion, and the corresponding products were not isolated. The loss of reactivity for **3i** and **3j** may be explained by complexation of the catalyst to the heteroatoms. The only open-chain saturated amine (Table 4, entry 10) showed nearly no conversion at all.

**Table 4:**
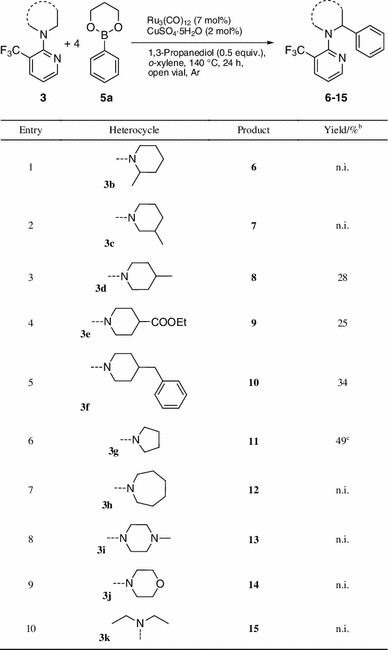
Scope of mono-arylations with different saturated cyclic amines **3** ^a^

^a^Reaction conditions: **3** (0.5 mmol), **5a** (2 mmol), Ru_3_(CO)_12_ (7 mol%), CuSO_4_·5H_2_O (2 mol%), 1,3-propanediol (0.25 mmol), 0.5 cm^3^ *o*-xylene, 140 °C, stirred for 24 h under argon conditions in an open vial

^b^Isolated yield after flash column chromatography; those examples with low or no conversion were not isolated (n.i.)

^c^Bis-arylated product was obtained in addition

**Fig. 1:**
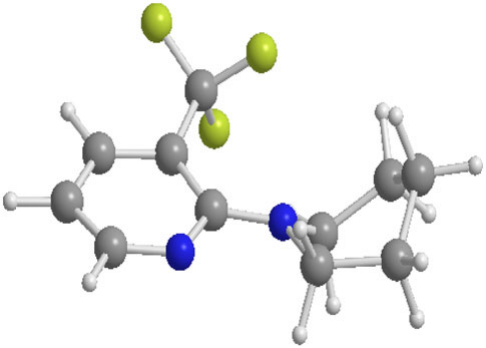
Optimized PBE1PBE geometry of the equilibrium structure of **3a**

As the reaction does not seem to be very tolerant regarding different rings, it was hypothesized that a specific geometry seems to be essential. Therefore, energy-minimization calculations were conducted for **3a**, **3b**, **3c**, **3d**, **3g**, **3i**, and **3j**. The six-membered rings, except for the 2-substituted piperidine **3b**, all have rather similar geometry according to the calculations. As shown in Fig. 1 for **3a**, they all have a slightly twisted boat conformation at the piperidine core. Especially the piperidine ring of **3a** and the 4-substituted piperidine ring of **3d** show very similar properties, and therefore the difference in yield could not be explained by the calculated geometries of these starting materials. A more conclusive result could have been obtained by calculating the corresponding Ru complexes formed during the reaction. However, since the nature of the active catalyst species is not known entirely, no such calculations could be conducted.

Finally, cleavage of the directing group was investigated. Initially, it was attempted to cleave the directing group via a method reported by Maes and coworkers [36]. They subjected the mono-arylated product (2-phenyl-1-pyridin-2-yl)piperidine to Pd-catalyzed hydrogenation (Pd/C, 1 atm H_2_, HCl, *i*-PrOH) and subsequent NH_2_NH_2_/AcOH treatment, which had already been used by Sames and coworkers [35], to cleave the pyrroline directing group. The same conditions were applied to 2-phenyl-1-(3-trifluoromethylpyridin-2-yl)piperidine (**4a**), but the reported protocol was not successful. It turned out that hydrogenation using Pd/C as catalyst was not working in presence of the CF_3_ group on pyridine. On changing the hydrogenation catalyst to PtO_2_ hydrate, almost pure intermediate **16** was obtained after basic extraction. For further purification, the crude product was dissolved in CH_2_Cl_2_ and silica gel was added before the solvent was evaporated. This procedure was undertaken to subject the compound to column chromatography as solid. Interestingly, not only the desired product 2-phenyl-1-[3-(trifluoromethyl)-3,4,5,6-tetrahydropyridin-2-yl]piperidine (**16**) could be obtained in yield of 12 % after purification, but also the product **17** lacking the trifluoromethyl group was isolated, in fact at much higher yield of 47 %. Obviously, this compound was generated during column chromatography, as it was not detected by GC–MS or ^1^H NMR of the crude product. Hence, intentional formation of **17** from **16** was attempted. Crude **16** was diluted in CH_2_Cl_2_ and stirred in the presence of approximately fivefold weight of silica gel in a closed vial at 50 °C. After 2.5 h, GC–MS showed full conversion to 2-phenyl-1-(3,4,5,6-tetrahydropyridin-2-yl)piperidine (**17**). To the best of our knowledge, this interesting detrifluoromethylation mediated by silica gel is unprecedented in the literature.

Both **16** (as crude material) and **17** were subjected to the cleavage conditions of NH_2_NH_2_/AcOH (2.5/0.7 M in EtOH) at 120 °C for 2 h in a closed vial, but only the reaction of **17** gave the desired product 2-phenylpiperidine (**18**) as detected by GC–MS and ^1^H NMR of the crude product after basic extraction. Also other reaction parameters (changing the acid to trifluoroacetic acid, increasing the reaction time or temperature) to cleave the reduced directing group from **16** did not lead to formation of **18**. Still, a new cleavage protocol was developed as shown in Scheme 3, providing the desired product **18** in 47 % yield. Hence, our deprotection protocol is competitive to the previously published removal procedure of the unsubstituted pyridine directing group, which was also cleaved in 47 % overall yield [36].

**Figure:**
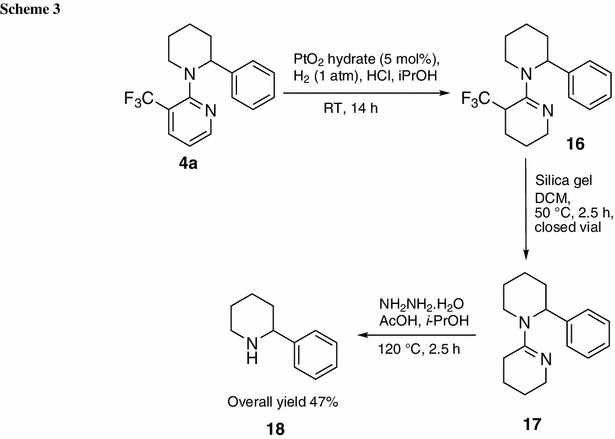

We propose the following mechanism for cleavage of the trifluoromethyl group (Scheme 4): Substrate **16** reacts with a free O–H group at the surface of silica gel to form **17**, and the triflouromethyl group is bound to silica gel. Next, water present in silica gel hydrolyzes the newly formed O–CF_3_ bond and trifluoromethanol is released, which then decomposes to carbonyl difluoride and hydrogen fluoride rapidly. This decomposition is described in the literature [41] to occur already at −20 °C, leading to gaseous compounds which can then not be detected using standard analytic techniques. The formation of gaseous compounds is supported by infrared (IR) analysis of the remaining silica gel after the deprotection, where no bands specific for C–F bonds were detected.

**Figure:**
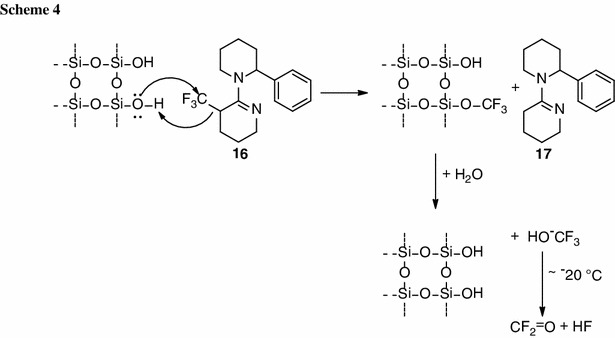

In conclusion, saturated cyclic amines could be mono-arylated in α-position by selective transition-metal-catalyzed C–H activation. Best results were obtained for piperidine. A number of different arylboronate esters were accepted in this reaction. For substituted piperidines the yields decreased and further dropped for all other saturated amines, with the exception of pyrrolidine. In this case, however, the different ring geometry led to some bis-arylation. Still, since purification problems between mono- and bis-arylated compounds can be avoided, the protocol offers significant potential in the synthesis of mono-arylated piperidines. For cleavage of the directing group, a modified protocol was developed, resulting in good yield. This interesting method takes advantage of an unprecedented CF_3_ cleavage under very mild conditions. Further exploration of the detrifluoromethylation may be of significant interest to the synthetic community and will be conducted in the near future.

## Experimental

Unless otherwise noted chemicals were purchased from commercial suppliers and were used without further purification. Flash column chromatography was performed on silica gel 60 from Merck (40–63 μm), using a Sepacore medium pressure liquid chromatography (MPLC) system from Büchi equipped with an ultraviolet (UV) light detector. TLC analysis was done with precoated aluminum-backed plates (silica gel 60 F254, Merck). Preparative TLC was performed on 20 × 20 cm, 1,000 μm thin-layer chromatography plates. Signals were visualized with UV light (254 nm). GC analyses were conducted on a Trace GC (Thermo Finnigan) using a BGB-5 (30 m × 0.32 mm i.d., 1.0 μm film thickness) polysiloxane (5 % diphenyl-, 95 % dimethylpolysiloxane) capillary column. The oven temperature program was 100 °C (2 min)/18 °C per min/280 °C (5 min). GC–MS analyses were conducted on a DSQ II GC–MS with Focus GC (Thermo Scientific), using a BGB-5 (30 m × 0.25 mm i.d., 0.25 μm film thickness) polysiloxane (5 % diphenyl-, 95 % dimethylpolysiloxane) capillary column. The oven temperature program was 100 °C (2 min)/18 °C per min/280 °C (3 min), unlike otherwise stated. Source and transfer line were set at 250 and 280 °C, respectively.

High-resolution mass spectroscopy measurements were carried out by E. Rosenberg at the Institute for Chemical Technologies and Analytics, Vienna University of Technology. All samples were analyzed by liquid chromatography (LC)-ion trap (IT)-time of flight (TOF)-MS with electrospray (ES) ionization and atmospheric-pressure chemical ionization (APCI) in positive ion detection mode, recording only MS^(1)^ spectra. The exact mass was used to calculate the elemental composition of the analytes from the quasimolecular ion [M + H]^+^ for the evaluation. Instrumental parameters: Shimadzu Prominence HPLC, consisting of: solvent degassing unit (DGU-20 A3), binary gradient pump (2× LC-20AD), auto-injector (SIL-20A), column oven (CTO-20AC), control module (CBM-20A), and diode array detector (SPD-M20A). Chromatography: column: Phenomenex Kinetex ODS(3), 30 × 4.6 mm, 2.6 μm core–shell particles, operated at 40 °C; gradient: 0 min: 70 % A, 30 % B (1 min); linear gradient to 5 min to 10 % A, 90 % B (hold until 10 min); at 10.01 min back to 70 % A, 30 % B (hold until 12.0 min); A: H_2_O (0.1 % v/v HCOOH), B: acetonitrile (0.1 % v/v HCOOH); flow: 0.5 cm^3^/min; injection volume: 0.5 mm^3^. MS parameters: MS parameters as in autotune. Data recorded with detector value at autotune value. Scan range: 100–1,000 amu for MS (PI) detection. ES ionization: curved desolvation line (CDL) temperature: 200 °C, heating block temperature: 200 °C.

Melting points were recorded using a Kofler-type Leica Galen III micro hot-stage microscope. Microwave reactions were performed on a BIOTAGE Initiator 60 microwave unit. The reported times are hold times. NMR spectra were recorded from CDCl_3_ solutions on a Bruker AC 200 (200 MHz) or a Bruker DRX 400 (400 MHz) spectrometer (as indicated), using the solvent peak [CDCl_3_: *δ* = 7.26 ppm (^1^H), *δ* = 77.16 ppm (^13^C)] and tetramethylsilane (TMS) as reference. ^13^C spectra were run in proton-decoupled mode, and in addition some spectra were also recorded as distortionless enhanced polarization transfer (DEPT) or attached proton test (APT).

### General procedure A

2-Chloro-3-(trifluoromethyl)pyridine (**1**, 1 equiv.), amine **2** (2 or 3 equiv.), K_2_CO_3_ (2 equiv.), and acetonitrile were placed in a microwave vial, a round-bottom flask with a reflux condenser, or a closed 8-cm^3^ vial, all equipped with a magnetic stirring bar (see respective compound for the reaction vessel used). The reaction mixture was heated, either in the microwave reactor or conventionally, and monitored by TLC and GC–MS. When reaction control showed full consumption of the starting material or no further progress, the mixture was cooled to room temperature and filtered, and the solvent was evaporated. The residue was purified by silica gel flash column chromatography to give the desired product **3**.

#### *1*-*(3*-*Trifluoromethylpyridin*-*2*-*yl)piperidine* (**3a**, C_11_H_13_F_3_N_2_)

Prepared according to general procedure A starting from 3.630 g **1** (20 mmol) and 3.406 g piperidine (**2a**, 40 mmol, 2 equiv.) using 40 cm^3^ acetonitrile. Conditions: round-bottom flask with reflux condenser, reflux, 24 h. Yield: 79 % (3.634 g); colorless oil; ^1^H NMR (CDCl_3_, 200 MHz): *δ* = 1.51–1.75 (m, 6H), 3.20–3.25 (m, 4H), 6.92 (dd, *J* = 7.7 Hz, 4.7 Hz, 1H), 7.83 (dd, *J* = 7.7 Hz, 1.7 Hz, 1H), 8.40 (br d, *J* = 3.7 Hz, 1H) ppm; ^13^C NMR (CDCl_3_, 50 MHz): *δ* = 24.4, 26.0, 51.9, 116.1, 116.6 (q, *J* = 31.3 Hz), 124.1 (q, *J* = 272.4 Hz), 137.2 (q, *J* = 5.1 Hz), 150.9, 160.5 ppm; HR-MS: [M + H]^+^ *m*/*z* (predicted) = 307.1417, *m*/*z* (measured) = 307.1409.

#### *2*-*Methyl*-*1*-*[3*-*(trifluoromethyl)pyridin*-*2*-*yl]piperidine* (**3b**, C_12_H_15_F_3_N_2_)

Prepared according to general procedure A starting from 363 mg **1** (2 mmol) and 594 mg 2-methylpiperidine (**2b**, 6 mmol, 3 equiv.) using 4 cm^3^ acetonitrile. Conditions: microwave, 180 °C, 3.5 h. Yield: 26 % (128.3 mg); slightly yellow oil; ^1^H NMR (CDCl_3_, 200 MHz): *δ* = 0.84 (d, 3H, *J* = 6.2 Hz), 1.33–1.84 (m, 6H), 2.74–2.86 (m, 1H), 2.99–3.09 (m, 1H), 3.36–3.52 (m, 1H), 7.12 (dd, *J* = 7.8 Hz, 4.8 Hz, 1H), 7.97 (dd, *J* = 7.8 Hz, 1.9 Hz, 1H), 8.57 (dd, *J* = 4.7 Hz, 1.2 Hz, 1H) ppm; ^13^C NMR (CDCl_3_, 50 MHz): *δ* = 19.3, 23.7, 26.0, 33.8, 53.6, 54.2, 119.0, 122.3 (q, *J* = 30.6 Hz), 123.5 (q, *J* = 272.4 Hz), 136.2 (q, *J* = 5.1 Hz), 151.6, 162.9 ppm; HR-MS: [M + H]^+^ *m*/*z* (predicted) = 245.1260, *m*/*z* (measured) = 245.1257.

#### *3*-*Methyl*-*1*-*[3*-*(trifluoromethyl)pyridin*-*2*-*yl]piperidine* (**3c**, C_12_H_15_F_3_N_2_)

Prepared according to general procedure A starting from 363 mg **1** (2 mmol) and 396 mg 3-methylpiperidine (**2c**, 4 mmol, 2 equiv.) using 4 cm^3^ acetonitrile. Conditions: microwave, 180 °C, 1.5 h. Yield: 87 % (423.6 mg); colorless oil; ^1^H NMR (CDCl_3_, 200 MHz): *δ* = 0.92 (d, 3H, *J* = 6.4 Hz), 0.97–1.17 (m, 1H), 1.59–1.89 (m, 4H), 2.53 (dd, *J* = 12.2 Hz, 10.2 Hz, 1H), 2.75–2.88 (m, 1H), 3.48–3.57 (m, 2H), 6.92 (dd, *J* = 7.7 Hz, 4.8 Hz, 1H), 7.83 (dd, *J* = 7.7 Hz, 1.8 Hz, 1H), 8.41 (dd, *J* = 4.7 Hz, 1.3 Hz, 1H) ppm; ^13^C NMR (CDCl_3_, 50 MHz): *δ* = 19.2, 25.4, 31.1, 33.0, 51.6, 58.5, 116.1, 116.5 (q, *J* = 31.2 Hz), 124.1 (q, *J* = 272.4 Hz), 137.2 (q, *J* = 5.1 Hz), 150.9, 160.3 ppm; HR-MS: [M + H]^+^ *m*/*z* (predicted) = 245.1260, *m*/*z* (measured) = 245.1252.

#### *4*-*Methyl*-*1*-*[3*-*(trifluoromethyl)pyridin*-*2*-*yl]piperidine* (**3d**, C_12_H_15_F_3_N_2_)

Prepared according to general procedure A starting from 363 mg **1** (2 mmol) and 396 mg 4-methylpiperidine (**2d**, 4 mmol, 2 equiv.) using 4 cm^3^ acetonitrile. Conditions: microwave, 180 °C, 1 h. Yield: 90 % (440.6 mg); colorless oil; ^1^H NMR (CDCl_3_, 200 MHz): *δ* = 0.99 (d, 3H, *J* = 6.1 Hz), 1.25–1.75 (m, 5H), 2.83–2.95 (m, 2H), 3.57–3.63 (m, 2H), 6.92 (dd, *J* = 7.7 Hz, 4.8 Hz, 1H), 7.83 (dd, *J* = 7.8 Hz, 1.8 Hz, 1H), 8.40 (br d, *J* = 3.6 Hz, 1H) ppm; ^13^C NMR (CDCl_3_, 50 MHz): *δ* = 21.9, 30.9, 34.3, 51.3, 116.1, 116.6 (q, *J* = 31.4 Hz), 124.1 (q, *J* = 272.4 Hz), 137.2 (q, *J* = 5.0 Hz), 150.8, 160.4 ppm; HR-MS: [M + H]^+^ *m*/*z* (predicted) = 245.1260, *m*/*z* (measured) = 245.1250.

#### *Ethyl 1*-*[3*-*(trifluoromethyl)pyridin*-*2*-*yl]piperidine*-*4*-*carboxylate* (**3e**, C_14_H_17_F_3_N_2_O_2_)

Prepared according to general procedure A starting from 363 mg **1** (2 mmol) and 628 mg ethyl piperidine-4-carboxylate (**2c**, 4 mmol, 2 equiv.) using 4 cm^3^ acetonitrile. Conditions: closed vial, 100 °C, 22.5 h. Yield: 75 % (454.5 mg); slightly yellow oil; ^1^H NMR (CDCl_3_, 200 MHz): *δ* = 1.28 (t, 3H, *J* = 7.1 Hz), 1.25–1.75 (m, 4H), 2.40–2.55 (m, 1H), 2.89–3.03 (m, 2H), 3.54–3.63 (m, 2H), 7.13 (q, 2H, *J* = 7.1 Hz), 6.98 (dd, *J* = 7.7 Hz, 4.8 Hz, 1H), 7.85 (dd, *J* = 7.8 Hz, 1.8 Hz, 1H), 8.42 (dd, *J* = 4.6 Hz, 1.4 Hz, 1H) ppm; ^13^C NMR (CDCl_3_, 50 MHz): *δ* = 13.9, 28.0, 40.9, 50.3, 60.1, 116.6, 117.0 (q, *J* = 31.4 Hz), 123.6 (q, *J* = 272.6 Hz), 136.9 (q, *J* = 5.0 Hz), 150.7, 159.9, 174.6 ppm; HR-MS: [M + H]^+^ *m*/*z* (predicted) = 303.1316, *m*/*z* (measured) = 303.1307.

#### *4*-*Benzyl*-*1*-*[3*-*(trifluoromethyl)pyridin*-*2*-*yl]piperidine* (**3f**, C_18_H_19_F_3_N_2_)

Prepared according to general procedure A starting from 363 mg **1** (2 mmol) and 700 mg 4-benzylpiperidine (**2f**, 4 mmol, 2 equiv.) using 4 cm^3^ acetonitrile. Conditions: closed vial, 100 °C, 22.5 h. Yield: 87 % (556 mg); slightly yellow solid; m.p.: 56–59 °C; ^1^H NMR (CDCl_3_, 200 MHz): *δ* = 1.32–1.76 (m, 5H), 2.60 (d, 2H, *J* = 6.6 Hz), 2.78–2.90 (m, 2H), 3.57–3.64 (m, 2H), 6.92 (dd, *J* = 7.7 Hz, 4.8 Hz, 1H), 7.16–7.34 (m, 5H), 7.82 (dd, *J* = 7.7 Hz, 1.8 Hz, 1H), 8.39 (dd, *J* = 4.6 Hz, 1.5 Hz, 1H) ppm; ^13^C NMR (CDCl_3_, 50 MHz): *δ* = 32.3, 38.0, 43.3, 51.2, 116.3, 116.6 (q, *J* = 31.4 Hz), 124.1 (q, *J* = 272.5 Hz), 128.2, 129.1, 137.2 (q, *J* = 5.1 Hz), 140.6, 150.9, 160.2 ppm; HR-MS: [M + H]^+^ *m*/*z* (predicted) = 321.1573, *m*/*z* (measured) = 321.1559.

#### *1*-*[3*-*(trifluoromethyl)pyridin*-*2*-*yl]pyrrolidine* (**3g**, C_10_H_11_F_3_N_2_)

Prepared according to general procedure A starting from 1.089 g **1** (6 mmol) and 853 mg pyrrolidine (**2g**, 12 mmol, 2 equiv.) using 12 cm^3^ acetonitrile. Conditions: round-bottom flask with reflux condenser, reflux, 48 h. Yield: 84 % (1.0884 g); colorless oil; ^1^H NMR (CDCl_3_, 200 MHz): *δ* = 1.88–2.01 (m, 4H), 3.56–3.62 (m, 4H), 6.62 (dd, *J* = 7.7 Hz, 4.7 Hz, 1H), 7.78 (dd, *J* = 7.8 Hz, 1.7 Hz, 1H), 8.27 (dd, *J* = 4.4 Hz, 1.2 Hz, 1H) ppm; ^13^C NMR (CDCl_3_, 50 MHz): *δ* = 25.6, 49.5 (q, *J* = 3.2 Hz), 108.6 (q, *J* = 32.2 Hz), 110.9, 124.5 (q, *J* = 271.2 Hz), 136.9 (q, *J* = 6.2 Hz), 150.6, 154.9 ppm; HR-MS: [M + H]^+^ *m*/*z* (predicted) = 217.0947, *m*/*z* (measured) = 217.0945.

#### *1*-*[3*-*(trifluoromethyl)pyridin*-*2*-*yl]hexahydroazepine* (**3h**, C_12_H_15_F_3_N_2_)

Prepared according to general procedure A starting from 1.452 g **1** (8 mmol) and 1.584 g hexahydroazepine (**2h**, 16 mmol, 2 equiv.) using 16 cm^3^ acetonitrile. Conditions: round-bottom flask with reflux condenser, reflux, 65 h. Yield: 86 % (1.6728 g); colorless oil; ^1^H NMR (CDCl_3_, 200 MHz): *δ* = 1.54–1.66 (m, 4H), 1.76–1.89 (m, 4H), 3.57 (t, *J* = 5.6 Hz, 4H), 6.72 (dd, *J* = 7.7 Hz, 4.6 Hz, 1H), 7.77 (dd, *J* = 7.8 Hz, 1.8 Hz, 1H), 8.29 (dd, *J* = 4.3 Hz, 1.3 Hz, 1H) ppm; ^13^C NMR (CDCl_3_, 200 MHz): *δ* = 27.6, 28.4, 51.9 (q, *J* = 2.2 Hz), 111.3 (q, *J* = 31.7 Hz), 116.3, 124.4 (q, *J* = 271.7 Hz), 137.5 (q, *J* = 5.7 Hz), 150.1, 158.8 ppm; HR-MS: [M + H]^+^ *m*/*z* (predicted) = 245.1260, *m*/*z* (measured) = 245.1250.

#### *1*-*Methyl*-*4*-*[3*-*(trifluoromethyl)pyridin*-*2*-*yl]piperazine* (**3i**, C_11_H_14_F_3_N_3_)

Prepared according to general procedure A starting from 1.452 g **1** (8 mmol) and 1.600 g 1-methylpiperazine (**2i**, 16 mmol, 2 equiv.) using 16 cm^3^ acetonitrile. Conditions: round-bottom flask with reflux condenser, reflux, 65 h. Yield: 83 % (1.632 g); slightly yellow oil; ^1^H NMR (CDCl_3_, 200 MHz): *δ* = 2.35 (s, 3H), 2.56 (t, *J* = 4.8 Hz, 4H), 3.35 (t, *J* = 4.8 Hz, 4H), 6.95 (dd, *J* = 7.7 Hz, 4.8 Hz, 1H), 7.84 (dd, *J* = 7.8 Hz, 1.5 Hz, 1H), 8.41 (br d, *J* = 4.8 Hz, 1H) ppm; ^13^C NMR (CDCl_3_, 50 MHz): *δ* = 46.1, 50.4, 55.1, 116.3 (q, *J* = 31.5 Hz), 116.4, 124.0 (q, *J* = 272.6 Hz), 137.2 (q, *J* = 5.1 Hz), 150.9, 159.4 ppm; HR-MS: [M + H]^+^ *m*/*z* (predicted) = 246.1213, *m*/*z* (measured) = 246.1200.

#### *1*-*[3*-*(trifluoromethyl)pyridin*-*2*-*yl]morpholine* (**3j**, C_10_H_11_F_3_N_2_O)

Prepared according to general procedure A starting from 182 mg **1** (1 mmol) and 174 mg morpholine (**2j**, 2 mmol, 2 equiv.) using 2 cm^3^ acetonitrile. Conditions: microwave, 180 °C, 1.5 h. Yield: 63 % (147.3 mg); colorless oil; ^1^H NMR (CDCl_3_, 200 MHz): *δ* = 3.29 (t, *J* = 4.6 Hz, 4H), 3.84 (t, *J* = 4.6 Hz, 4H), 7.01 (dd, *J* = 7.8 Hz, 4.8 Hz, 1H), 7.89 (dd, *J* = 7.8 Hz, 1.7 Hz, 1H), 8.44 (br d, *J* = 4.3 Hz, 1H) ppm; ^13^C NMR (CDCl_3_, 50 MHz): *δ* = 51.1, 66.9, 117.0 (q, *J* = 31.5 Hz), 117.1, 123.9 (q, *J* = 272.5 Hz), 137.2 (q, *J* = 5.1 Hz), 151.1, 159.5 ppm; HR-MS: [M + H]^+^ *m*/*z* (predicted) = 233.0896, *m*/*z* (measured) = 233.0888.

#### *N*-*Ethyl*-*N*-*[3*-*(trifluoromethyl)pyridin*-*2*-*yl]ethanamine* (**3k**, C_10_H_13_F_3_N_2_)

Prepared according to general procedure A starting from 363 mg **1** (2 mmol) and 439 mg *N*-ethylethanamine (**2k**, 6 mmol, 3 equiv.) using 4 cm^3^ acetonitrile. Conditions: microwave, 180 °C, 2 h. Yield: 37 % (160.8 mg); slightly yellow oil; ^1^H NMR (CDCl_3_, 200 MHz): *δ* = 1.09 (t, 6H, *J* = 7.1 Hz), 3.30 (q, 4H, *J* = 7.1 Hz), 6.93 (dd, *J* = 7.8 Hz, 4.7 Hz, 1H), 7.85 (dd, *J* = 7.8 Hz, 1.8 Hz, 1H), 8.42 (dd, *J* = 4.6 Hz, 1.2 Hz, 1H) ppm; ^13^C NMR (CDCl_3_, 50 MHz): *δ* = 12.8, 46.1, 116.1, 117.6 (q, *J* = 31.1 Hz), 124.0 (q, *J* = 272.2 Hz), 137.0 (q, *J* = 5.3 Hz), 150.6, 159.9 ppm; HR-MS: [M + H]^+^ *m*/*z* (predicted) = 219.1104, *m*/*z* (measured) = 219.1009.

### General procedure B

An 8-cm^3^ vial with a magnetic stirring bar and a screw cap with septum was charged with saturated cyclic amine **3** (0.5 mmol, 1 equiv.), arylboronate ester **5** (2 mmol, 4 equiv.), 22 mg Ru_3_(CO)_12_ (0.035 mmol, 7 mol%), 19 mg 1,3-propanediol (0.25 mmol, 0.5 equiv.), and 2.5 mg CuSO_4_·5H_2_O (0.01 mmol, 2 mol%). The vial was evacuated and flushed with argon three times, and 0.5 cm^3^ *o*-xylene was added via syringe. Then, the vial was equipped with a balloon filled with argon, which was attached to a needle and plunged through the septum. The mixture was heated to 140 °C for 24 h in a heating block with a reflux condenser block on top. The reaction mixture was cooled to room temperature, and 2 cm^3^ EtOAc and 2 cm^3^ water were added to the reaction solution and agitated. The mixture was extracted three times with EtOAc. To achieve better phase separation, brine was added if necessary. The combined organic layers were dried over Na_2_SO_4_ and filtered, and the solvent was evaporated. The residue was purified by silica gel flash chromatography to give **4**.

#### *2*-*Phenyl*-*1*-*[3*-*(trifluoromethyl)pyridin*-*2*-*yl]piperidine* (**4a**, C_17_H_17_F_3_N_2_)

Yield: 60 % (91.1 mg); colorless solid; m.p.: 92–94 °C; ^1^H NMR (CDCl_3_, 400 MHz): *δ* = 1.52–1.61 (m, 1H), 1.72–1.90 (m, 5H), 2.77 (ddd, *J* = 11.5 Hz, 11.5 Hz, 1.7 Hz, 1H), 3.32–3.36 (m, 1H), 4.46 (br d, *J* = 10.0 Hz, 1H), 6.89 (dd, *J* = 7.2 Hz, 5.0 Hz, 1H), 6.98–7.02 (m, 1H), 7.09 (t, *J* = 7.6 Hz, 2H), 7.31 (d, *J* = 7.9 Hz, 2H), 7.75 (dd, *J* = 7.8 Hz, 1.4 Hz, 1H), 8.32 (br d, *J* = 4.7 Hz, 1H) ppm; ^13^C NMR (CDCl_3_, 100 MHz): *δ* = 24.9, 26.1, 35.6, 56.7, 64.3, 118.9, 121.7 (q, *J* = 30.9 Hz), 123.5 (q, *J* = 272.6 Hz), 126.2, 127.7, 127.9, 136.3 (q, *J* = 5.2 Hz), 144.3, 151.1, 162.5 ppm; HR-MS: [M + H]^+^ *m*/*z* (predicted) = 307.1417, *m*/*z* (measured) = 307.1409.

#### *2*-*(p*-*Tolyl)*-*1*-*[3*-*(trifluoromethyl)pyridin*-*2*-*yl]piperidine* (**4b**, C_18_H_19_F_3_N_2_)

Yield: 47 % (84.8 mg); slightly yellow solid; m.p.: 61–63 °C; ^1^H NMR (CDCl_3_, 400 MHz): *δ* = 1.52–1.57 (m, 1H), 1.71–1.90 (m, 5H), 2.17 (s, 3H), 2.76 (ddd, *J* = 11.5 Hz, 11.5 Hz, 2.5 Hz, 1H), 3.30–3.34 (m, 1H), 4.43 (dd, *J* = 10.1 Hz, 2.8 Hz, 1H), 6.88–6.91 (m, 3H), 7.19 (d, *J* = 7.9 Hz, 2H), 7.75 (dd, *J* = 7.8 Hz, 1.7 Hz, 1H), 8.34 (dd, *J* = 4.8 Hz, 1.5 Hz, 1H) ppm; ^13^C NMR (CDCl_3_, 100 MHz): *δ* = 21.0, 24.9, 26.1, 35.6, 56.7, 64.0, 118.9, 121.6 (q, *J* = 30.9 Hz), 123.5 (q, *J* = 272.7 Hz,), 127.6, 127.8, 135.6, 136.3 (q, *J* = 5.2 Hz), 141.3, 151.1, 162.7 ppm; HR-MS: [M + H]^+^ *m*/*z* (predicted) = 321.1557, *m*/*z* (measured) = 321.1564.

#### *2*-*(4*-*tert*-*Butylphenyl)*-*1*-*[3*-*(trifluoromethyl)pyridin*-*2*-*yl]piperidine* (**4c**, C_21_H_25_F_3_N_2_)

Yield: 50 % (90.7 mg); colorless solid; m.p.: 93–96 °C; ^1^H NMR (CDCl_3_, 200 MHz): *δ* = 1.20 (s, 9H), 1.51–1.90 (m, 6H), 2.75–2.87 (m, 1H), 3.28–3.39 (m, 1H), 4.46–4.53 (m, 1H), 6.89 (dd, *J* = 7.7 Hz, 4.9 Hz, 1H), 7.09–7.24 (m, 4H), 7.76 (br d, *J* = 6.7 Hz, 1H), 8.34 (br d, *J* = 3.6 Hz, 1H) ppm; ^13^C NMR (CDCl_3_, 50 MHz): *δ* = 24.6, 26.1, 31.3, 34.2, 35.3, 56.2, 63.6, 115.5, 121.2 (q, *J* = 30.8 Hz), 123.6 (q, *J* = 272.7 Hz), 124.5, 127.4, 136.3 (q, *J* = 5.2 Hz), 141.0, 148.7, 151.1, 162.5 ppm; HR-MS: [M + H]^+^ *m*/*z* (predicted) = 363.2043, *m*/*z* (measured) = 263.2036.

#### *2*-*(4*-*Fluorophenyl)*-*1*-*[3*-*(trifluoromethyl)pyridin*-*2*-*yl]piperidine* (**4d**, C_17_H_16_F_4_N_2_)

Yield: 43 % (68.9 mg); slightly yellow solid; m.p.: 70–72 °C; ^1^H NMR (CDCl_3_, 200 MHz): *δ* = 1.46–1.94 (m, 6H), 2.75 (ddd, *J* = 11.2 Hz, 11.2 Hz, 3.1 Hz, 1H), 3.28–3.38 (m, 1H), 4.43 (dd, *J* = 9.7 Hz, 3.3 Hz, 1H), 6.73–6.83 (m, 2H), 6.95 (dd, *J* = 7.8 Hz, 4.8 Hz, 1H), 7.23–7.30 (m, 2H), 7.78 (dd, *J* = 7.8 Hz, 1.6 Hz, 1H), 8.35 (dd, *J* = 4.7 Hz, 1.4 Hz, 1H) ppm; ^13^C NMR (CDCl_3_, 50 MHz): *δ* = 24.8, 26.0, 35.6, 56.8, 63.6, 114.4 (d, *J* = 21.4 Hz), 119.2, 121.9 (q, *J* = 30.8 Hz), 123.4 (q, *J* = 272.7 Hz), 129.4 (d, *J* = 7.7 Hz), 136.2 (q, *J* = 5.3 Hz), 140.0 (d, *J* = 3.2 Hz), 151.1, 161.2 (d, *J* = 243.7 Hz), 162.5 ppm; HR-MS: [M + H]^+^ *m*/*z* (predicted) = 325.1322, *m*/*z* (measured) = 325.1317.

#### *2*-*(4*-*Chlorophenyl)*-*1*-*[3*-*(trifluoromethyl)pyridin*-*2*-*yl]piperidine* (**4e**, C_17_H_16_ClF_3_N_2_)

Yield: 34 % (58.0 mg); colorless oil; ^1^H NMR (CDCl_3_, 400 MHz): *δ* = 1.48–1.60 (m, 1H), 1.72–1.91 (m, 5H), 2.73 (ddd, *J* = 11.6 Hz, 11.6 Hz, 2.5 Hz, 1H), 3.31–3.36 (m, 1H), 4.42 (dd, *J* = 10.8 Hz, 2.5 Hz, 1H), 6.94 (dd, *J* = 7.7 Hz, 4.9 Hz, 1H), 7.06 (d, *J* = 8.3 Hz, 2H), 7.24 (d, *J* = 8.5 Hz, 2H), 7.78 (dd, *J* = 7.8 Hz, 1.7 Hz, 1H), 8.34 (dd, *J* = 4.7 Hz, 1.5 Hz, 1H) ppm; ^13^C NMR (CDCl_3_, 100 MHz): *δ* = 24.8, 26.0, 35.6, 56.9, 63.7, 119.2, 121.8 (q, *J* = 30.9 Hz), 123.4 (q, *J* = 272.7 Hz), 127.9, 129.3, 131.7, 136.3 (q, *J* = 5.2 Hz), 142.9, 151.2, 162.3 ppm; HR-MS: [M + H]^+^ *m*/*z* (predicted) = 341.1027, *m*/*z* (measured) = 341.1035.

#### *2*-*(4*-*Methoxyphenyl)*-*1*-*[3*-*(trifluoromethyl)pyridin*-*2*-*yl]piperidine* (**4f**, C_18_H_19_F_3_N_2_O)

Yield: 16 % (26.5 mg); slightly yellow solid; m.p.: 69–72 °C; ^1^H NMR (CDCl_3_, 200 MHz): *δ* = 1.46–1.91 (m, 6H), 2.76 (ddd, *J* = 11.3 Hz, 11.3 Hz, 3.1 Hz, 1H), 3.26–3.36 (m, 1H), 3.68 (s, 3H), 4.40 (dd, *J* = 9.1 Hz, 3.8 Hz, 1H), 6.64 (d, *J* = 8.6 Hz, 2H), 6.93 (dd, *J* = 7.8 Hz, 4.8 Hz, 1H), 7.22 (d, *J* = 8.6 Hz, 2H), 7.77 (dd, *J* = 7.8 Hz, 1.6 Hz, 1H), 8.36 (br d, *J* = 3.8 Hz, 1H) ppm; ^13^C NMR (CDCl_3_, 50 MHz): *δ* = 24.9, 26.1, 35.5, 55.0, 56.7, 63.6, 113.0, 119.0, 121.8 (q, *J* = 30.8 Hz), 123.5 (q, *J* = 272.7 Hz), 129.1, 136.2 (qd, ^*3*^ *J* = 5.2 Hz), 136.4, 151.1, 157.8, 162.7 ppm; HR-MS: [M + H]^+^ *m*/*z* (predicted) = 337.1522, *m*/*z* (measured) = 337.1515.

#### *2*-*(4*-*Trifluoromethylphenyl)*-*1*-*[3*-*(trifluoromethyl)pyridin*-*2*-*yl]piperidine* (**4g**, C_18_H_16_F_6_N_2_)

Yield: 40 % (74.9 mg); slightly yellow solid; m.p.: 60–62 °C; ^1^H NMR (CDCl_3_, 400 MHz): *δ* = 1.53–1.62 (m, 1H), 1.73–1.93 (m, 5H), 2.74 (ddd, *J* = 11.6 Hz, 11.6 Hz, 2.5 Hz, 1H), 3.35–3.40 (m, 1H), 4.53 (dd, *J* = 10.9 Hz, 2.4 Hz, 1H), 6.95 (dd, *J* = 7.7 Hz, 4.9 Hz, 1H), 7.35 (d, *J* = 8.3 Hz, 2H), 7.42 (d, *J* = 8.2 Hz, 2H), 7.80 (dd, *J* = 7.8 Hz, 1.7 Hz, 1H), 8.32 (dd, *J* = 4.8 Hz, 1.4 Hz, 1H) ppm; ^13^C NMR (CDCl_3_, 100 MHz): *δ* = 24.7, 26.0, 35.6, 56.9, 64.0, 119.3, 121.7 (q, *J* = 30.9 Hz), 123.4 (q, *J* = 272.8 Hz), 124.2 (q, *J* = 271.8 Hz), 124.8 (q, *J* = 3.8 Hz), 128.1, 128.4 (q, *J* = 32.3 Hz), 136.5 (q, *J* = 5.2 Hz), 148.6, 151.1, 162.0 ppm; HR-MS: [M + H]^+^ *m*/*z* (predicted) = 375.1290, *m*/*z* (measured) = 375.1280.

#### *2*-*(m*-*Tolyl)*-*1*-*[3*-*(trifluoromethyl)pyridin*-*2*-*yl]piperidine* (**4j**, C_18_H_19_F_3_N_2_)

Yield: 49 % (78.8 mg) colorless solid; m.p.: 80–82 °C; ^1^H NMR (CDCl_3_, 200 MHz): *δ* = 1.47–1.88 (m, 6H), 2.19 (s, 3H), 2.73 (ddd, *J* = 11.3 Hz, 11.3 Hz, 2.9 Hz, 1H), 3.27–3.38 (m, 1H), 4.43 (dd, *J* = 9.3 Hz, 3.3 Hz, 1H), 6.79–7.16 (m, 5H), 7.75 (dd, *J* = 7.8 Hz, 1.6 Hz, 1H), 8.33 (dd, *J* = 4.8 Hz, 1.3 Hz, 1H) ppm; ^13^C NMR (CDCl_3_, 50 MHz): *δ* = 21.3, 24.8, 26.1, 35.6, 56.7, 64.2, 118.9, 121.6 (q, *J* = 30.9 Hz), 123.5 (q, *J* = 272.7 Hz), 124.9, 126.9, 127.5, 128.6, 136.3 (q, *J* = 5.2 Hz), 137.1, 144.3, 151.1, 162.6 ppm; HR-MS: [M + H]^+^ *m*/*z* (predicted) = 321.1573, *m*/*z* (measured) = 321.1569.

#### *2*-*(3*-*Chlorophenyl)*-*1*-*[3*-*(trifluoromethyl)pyridin*-*2*-*yl]piperidine* (**4k**, C_17_H_16_ClF_3_N_2_)

Yield: 39 % (66.6 mg); colorless solid; m.p.: 82–84 °C; ^1^H NMR (CDCl_3_, 200 MHz): *δ* = 1.45–1.92 (m, 6H), 2.72 (ddd, *J* = 11.2 Hz, 11.2 Hz, 3.1 Hz, 1H), 3.29–3.39 (m, 1H), 4.45 (dd, *J* = 10.1 Hz, 2.5 Hz, 1H), 6.92–7.06 (m, 3H), 7.15–7.26 (m, 1H), 7.33 (br s, 1H), 7.80 (dd, *J* = 7.8 Hz, 1.4 Hz, 1H), 8.35 (dd, *J* = 4.8 Hz, 1.2 Hz, 1H) ppm; ^13^C NMR (CDCl_3_, 100 MHz): *δ* = 24.7, 26.0, 35.5, 56.8, 63.8, 119.2, 121.7 (q, *J* = 30.9 Hz), 123.5 (q, *J* = 272.6 Hz), 126.1, 126.4, 128.1, 129.0, 133.5, 136.4 (q, *J* = 5.1 Hz), 146.5, 151.2, 162.2 ppm; HR-MS: [M + H]^+^ *m*/*z* (predicted) = 341.1027, *m*/*z* (measured) = 341.1017.

#### *4*-*Methyl*-*2*-*phenyl*-*1*-*[3*-*(trifluoromethyl)pyridin*-*2*-*yl]piperidine* (**8**, C_18_H_19_F_3_N_2_)

Yield: 28 % (44.4 mg); colorless solid; m.p.: 106–108 °C; ^1^H NMR (CDCl_3_, 400 MHz): *δ* = 1.00 (d, *J* = 6.2 Hz, 3H), 1.44–1.61 (m, 2H), 1.68–1.78 (m, 2H), 1.84–1.89 (m, 1H), 2.75 (ddd, *J* = 11.9 Hz, 11.9 Hz, 1.8 Hz, 1H), 3.43 (ddd, *J* = 11.7 Hz, 11.7 Hz, 3.1 Hz, 1H), 4.42 (dd, *J* = 11.5 Hz, 1.9 Hz, 1H), 6.92 (dd, *J* = 7.6 Hz, 4.9 Hz, 1H), 7.06–7.10 (m, 1H), 7.08 (br dd, *J* = 7.5 Hz, 7.5 Hz, 2H), 7.30 (br d, *J* = 7.5 Hz, 2H), 7.76 (dd, *J* = 7.8 Hz, 1.6 Hz, 1H), 8.33 (dd, *J* = 4.7 Hz, 1.4 Hz, 1H) ppm; ^13^C NMR (CDCl_3_, 100 MHz): *δ* = 22.0, 31.7, 34.5, 44.7, 57.0, 64.4, 119.3, 122.3 (q, *J* = 30.6 Hz), 123.4 (q, *J* = 272.9 Hz), 126.2, 127.7, 127.9, 136.2 (q, *J* = 5.1 Hz), 144.4, 151.1, 162.7 ppm; HR-MS: [M + H]^+^ *m*/*z* (predicted) = 321.1573, *m*/*z* (measured) = 321.1565.

#### *Ethyl 2*-*phenyl*-*1*-*[3*-*(trifluoromethyl)pyridin*-*2*-*yl]piperidine*-*4*-*carboxylate* (**9**, C_20_H_21_F_3_N_2_O_2_)

Yield: 25 % (47.6 mg); slightly yellow solid; m.p.: 74–76 °C; ^1^H NMR (CDCl_3_, 200 MHz): *δ* = 1.25 (t, *J* = 7.1 Hz, 3H), 1.86–2.21 (m, 4H), 2.55–2.84 (m, 2H), 3.43 (ddd, *J* = 11.7 Hz, 11.7 Hz, 3.4 Hz, 1H), 4.14 (q, *J* = 7.1 Hz, 2H), 4.46 (dd, *J* = 11.3 Hz, 2.4 Hz, 1H), 6.92–7.14 (m, 3H), 7.29–7.34 (m, 2H), 7.78 (dd, *J* = 7.8 Hz, 1.5 Hz, 1H), 8.34 (dd, *J* = 4.5 Hz, 1.2 Hz, 1H) ppm; ^13^C NMR (CDCl_3_, 100 MHz): *δ* = 14.2, 28.5, 37.9, 42.2, 56.0, 63.8, 119.7, 122.4 (q, *J* = 31.0 Hz), 123.4 (q, *J* = 272.8 Hz), 126.6, 127.8, 128.0, 136.3 (q, *J* = 5.1 Hz), 141.2, 151.1, 162.1 ppm; HR-MS: [M + H]^+^ *m*/*z* (predicted) = 379.1628, *m*/*z* (measured) = 379.1629.

#### *4*-*Benzyl*-*2*-*phenyl*-*1*-*[3*-*(trifluoromethyl)pyridin*-*2*-*yl]piperidine* (**10**, C_24_H_23_F_3_N_2_)

Yield: 34 % (67.8 mg); slightly yellow gum; ^1^H NMR (CDCl_3_, 200 MHz): *δ* = 1.46–1.93 (m, 5H), 2.59–2.76 (m, 2H), 3.35 (ddd, *J* = 11.7 Hz, 11.7 Hz, 3.2 Hz, 1H), 4.39 (bd, *J* = 9.8 Hz, 1H), 6.87–7.31 (m, 11H), 7.75 (dd, *J* = 7.8 Hz, 1.6 Hz, 1H), 8.31 (dd, *J* = 4.7 Hz, 1.3 Hz, 1H) ppm; ^13^C NMR (CDCl_3_, 100 MHz): *δ* = 32.4, 38.8, 42.5, 43.4, 56.8, 64.3, 119.4, 122.3 (q, *J* = 30.8 Hz), 123.5 (q, *J* = 272.7 Hz), 125.9, 126.3, 127.7, 128.0, 128.3, 129.2, 136.2 (q, *J* = 5.1 Hz), 140.5, 144.2, 151.1, 162.5 ppm; HR-MS: [M + H]^+^ *m*/*z* (predicted) = 397.1886, *m*/*z* (measured) = 397.1888.

#### *2*-*Phenyl*-*1*-*[3*-*(trifluoromethyl)pyridin*-*2*-*yl]pyrrolidine* (**11**, C_16_H_15_F_3_N_2_)

Yield: 49 % (72.2 mg); slightly yellow solid; m.p.: 81–84 °C; ^1^H NMR (CDCl_3_, 400 MHz): *δ* = 1.82–2.07 (m, 3H), 2.37–2.42 (m, 1H), 3.57–3.61 (m, 1H), 3.92–3.98 (m, 1H), 5.54 (dd, *J* = 7.8 Hz, 7.8 Hz, 1H), 6.61 (dd, *J* = 7.7 Hz, 4.7 Hz, 1H), 7.13–7.16 (m, 1H), 7.24 (t, *J* = 7.5 Hz, 2H), 7.30 (d, *J* = 7.9 Hz, 2H), 7.74 (dd, *J* = 7.7 Hz, 1.2 Hz, 1H), 8.32 (br d, *J* = 4.4 Hz, 1H) ppm; ^13^C NMR (CDCl_3_, 100 MHz): *δ* = 26.0, 35.9, 52.5 (q, ^*5*^ *J* = 5.2 Hz), 63.1, 111.1 (q, *J* = 30.9 Hz), 112.6, 123.2 (q, *J* = 271.7 Hz), 126.1, 126.3, 128.2, 136.9 (q, *J* = 5.7 Hz), 144.9, 150.4, 155.5 ppm; HR-MS: [M + H]^+^ *m*/*z* (predicted) = 293.1260, *m*/*z* (measured) = 293.1249.

#### *2*-*Phenyl*-*1*-*(3,4,5,6*-*tetrahydropyridin*-*2*-*yl)piperidine* (**17**, C_16_H_22_N_2_)

PtO_2_·aq (16.3 mg, 0.072 mmol, 5 mol%) was placed in a three-necked 25-cm^3^ flask equipped with a magnetic stirrer. The flask was evacuated and flushed with nitrogen three times, and then 7.2 cm^3^ *i*-PrOH was added with a syringe through a septum. After stirring the mixture at room temperature (RT) for some minutes, a solution of 441 mg **4a** (1.44 mmol, 1 equiv.) in 7.2 cm^3^ *i*-PrOH and 1.4 cm^3^ 2 N HCl was added to the catalyst with a syringe. The flask was flushed with hydrogen two times, and a hydrogen balloon was attached. The reaction mixture was then stirred at RT for 14 h and filtered through a pad of Celite^®^, and the solvent was evaporated. The residue was taken up in 1 N NaOH solution and subsequently extracted with CH_2_Cl_2_ three times. The combined organic layers were dried over Na_2_SO_4_, filtered, and evaporated to dryness. The crude product was then diluted in 5 cm^3^ CH_2_Cl_2_ and stirred in the presence of 1.6 g silica gel in a closed vial at 50 °C for 2.5 h. Afterwards, the crude product was directly rotated onto the silica gel and purified by silica gel flash column chromatography. Yield: 72 % (252 mg); colorless oil; ^1^H NMR (CDCl_3_, 200 MHz): *δ* = 1.36–2.41 (m, 12H), 2.75–2.87 (m, 1H), 3.43–3.66 (m, 2H), 4.12 (br d, *J* = 13.3 Hz, 1H), 5.36 (br s, 1H), 7.16–7.36 (m, 5H) ppm; ^13^C NMR (CDCl_3_, 200 MHz): *δ* = 19.7, 21.0, 22.6, 24.8, 25.5, 29.0, 40.0, 47.2, 52.8, 126.1, 126.7, 128.5, 141.6, 155.7 ppm; HR-MS: [M + H]^+^ *m*/*z* (predicted) = 243.1856, *m*/*z* (measured) = 243.1846.

#### *2*-*Phenylpiperidine* (**18**)

A three-necked 25-cm^3^ flask, equipped with a magnetic stirrer, was charged with 5.7 mg PtO_2_ hydrate (0.025 mmol, 5 mol%), evacuated, and flushed with nitrogen three times. Then, 2.5 cm^3^ *i*-PrOH was added with a syringe through a septum, and the mixture was stirred at RT. After some minutes, a solution of 153 mg **4a** (0.5 mmol, 1 equiv.) in 2.5 cm^3^ *i*-PrOH and 0.5 cm^3^ 2 N HCl was added to the catalyst with a syringe. The flask was flushed with hydrogen two times, and a hydrogen balloon was attached. The reaction mixture was then stirred at RT for 14 h and filtered through a pad of Celite^®^, and the solvent was evaporated. The residue was taken up in 1 N NaOH solution and subsequently extracted with CH_2_Cl_2_ three times. The combined organic layers were dried over Na_2_SO_4_, filtered, and evaporated to dryness. The crude product **16** was then diluted in 2.5 cm^3^ CH_2_Cl_2_ and stirred in the presence of 700 mg silica gel in a closed vial at 50 °C for 2.5 h. The crude intermediate **17** was eluted from the silica gel with a mixture of EtOAc and Et_3_N (15:1, then 1:1), evaporated to dryness, and then treated with 2.5 cm^3^ NH_2_NH_2_/AcOH (2.5 M/0.7 M in EtOH) at 120 °C for 2 h in a closed microwave vial under nitrogen atmosphere. Again the solvent was evaporated, and the residue was brought in 2 N NaOH solution and subsequently extracted with Et_2_O three times. Evaporation to dryness yielded the crude product. This was further purified by silica gel flash column chromatography, affording 37.9 mg (47 %) **18** as colorless gum. NMR spectra were found to be in accordance with the ones described in reference [36].

### Computational details

All calculations were performed using the Gaussian09 software package on the Phoenix Linux Cluster of the Vienna University of Technology [42]. The geometry and energy of the model compounds were optimized at the PBE1PBE level [43–45] with the 6-31G** basis set employed for all atoms [46–52]. All geometries were optimized without symmetry constraints. Frequency calculations were performed to confirm the nature of the stationary points, yielding no imaginary frequencies.

## References

[1] Bergmann RG (2007). Nature.

[2] Sames D, Godula K (2006). Science.

[3] Labinger JA, Bercaw JE (2002). Nature.

[4] Shilov AE, Shulpin GB (1997). Chem Rev.

[5] Yu JQ, Shi Z (2010). C–H activation.

[6] Dyker G (2005). Handbook of C–H transformations.

[7] Schnürch M, Dastbaravardeh N, Ghobrial M, Mrozek B, Mihovilovic MD (2011). Curr Org Chem.

[8] Ackermann L, Vicente R, Kapdi AR (2009). Angew Chem Int Ed.

[9] Ackermann L, Diers E, Manvar A (2012). Org Lett.

[10] Wencel-Delord J, Nimphius C, Patureau FW, Glorius F (2012). Angew Chem Int Ed.

[11] Flegeau EF, Bruneau C, Dixneuf PH, Jutand A (2011). J Am Chem Soc.

[12] Shiota H, Ano Y, Aihara Y, Fukumoto Y, Chatani N (2011). J Am Chem Soc.

[13] Tauchert ME, Incarvito CD, Rheingold AL, Bergman RG, Ellman JA (2012). J Am Chem Soc.

[14] Koley M, Dastbaravardeh N, Schnürch M, Mihovilovic MD (2012). Chem Cat Chem.

[15] Kakiuchi F, Chatani N (2003). Adv Synth Catal.

[16] Fairlamb IJS (2007). Annu Rep Prog Chem Sect B Org Chem.

[17] McGlacken GP, Bateman LM (2009). Chem Soc Rev.

[18] Rousseaux S, Gorelsky SI, Chung BKW, Fagnou K (2010). J Am Chem Soc.

[19] Pan S, Endo K, Shibata T (2011). Org Lett.

[20] Sundararaju B, Achard M, Sharma GVM, Bruneau C (2011). J Am Chem Soc.

[21] Jazzar R, Hitce J, Renaudat A, Sofack-Kreutzer J, Baudoin O (2010). Chem Eur J.

[22] Shabashov D, Daugulis O (2005). Org Lett.

[23] Murahashi SI, Komiya N, Terai H (2005). Angew Chem Int Ed.

[24] Tsuchikama K, Kasagawa M, Endo K, Shibata T (2009). Org Lett.

[25] Chaumontet M, Piccardi R, Baudoin O (2009). Angew Chem Int Ed.

[26] Bellina F, Rossi R (2010). Chem Rev.

[27] Sundararaju B, Tang Z, Achard M, Sharma GVM, Toupet L, Bruneau C (2010). Adv Synth Catal.

[28] Ghobrial M, Harhammer K, Mihovilovic MD, Schnürch M (2010). Chem Commun.

[29] Baudoin O (2011). Chem Soc Rev.

[30] Wasa M, Engle KM, Lin DW, Yoo EJ, Yu JQ (2011). J Am Chem Soc.

[31] Ghobrial M, Schnürch M, Mihovilovic MD (2011). J Org Chem.

[32] Guo Z, Orth P, Zhu Z, Mazzola RD, Chan TY, Vaccaro HA, Mc Kittrick B, Kozlowski JA, Lavey BJ, Zhou G, Paliwal S, Wong S-C, Shih N-Y, Ting PC, Rosner KE, Shipps GW, Siddiqui MA, Belanger DB, Dai C, Li D, Girijavallabhan VM, Popovici-Muller J, Yu W, Zhao L (2005). Preparation of tartaric acid functional compounds for the treatment of inflammatory disorders. Chem Abstr.

[33] Scott JD, Weinstein J, Miller MW, Stamford AW, Gilbert EJ, Xia Y, Greenlee WJ, Li W (2007). Diaryl piperidines as CB1 modulators and their preparation, pharmaceutical compositions and use in the treatment of diseases. Chem Abstr.

[34] Campos KR (2007). Chem Soc Rev.

[35] Pastine SJ, Gribkov DV, Sames D (2006). J Am Chem Soc.

[36] Prokopcova H, Bergman SD, Aelvoet K, Smout V, Herrebout W, Van der Veken B, Meerpoel L, Maes BUW (2010). Chem Eur J.

[37] Dastbaravardeh N, Schnürch M, Mihovilovic MD (2012). Org Lett.

[38] Dastbaravardeh N, Kirchner K, Schnürch M, Mihovilovic MD (2013). J Org Chem.

[39] Dastbaravardeh N, Schnürch M, Mihovilovic MD (2012). Org Lett.

[40] Schnürch M, Holzweber M, Mihovilovic MD, Stanetty P (2007). Green Chem.

[41] Seppelt K (1977). Angew Chem Int Ed.

[42] Frisch MJ, Trucks GW, Schlegel HB, Scuseria GE, Robb MA, Cheeseman, Scalmani G, Barone V, Mennucci B, Petersson GA, Nakatsuji H, Caricato M, Li X, Hratchian HP, Izmaylov AF, Bloino J, Zheng G, Sonnenberg JL, Hada M, Ehara M, Toyota K, Fukuda R, Hasegawa J, Ishida M, Nakajima T, Honda Y, Kitao O, Nakai H, Vreven T, Montgomery JA, Peralta JE, Ogliaro F, Bearpark M, Heyd JJ, Brothers E, Kudin KN, Staroverov VN, Kobayashi R, Normand J, Raghavachari K, Rendell A, Burant JC, Iyengar SS, Tomasi J, Cossi M, Rega N, Millam JM, Klene M, Knox JE, Cross JB, Bakken V, Adamo C, Jaramillo J, Gomperts R, Stratmann RE, Yazyev O, Austin AJ, Cammi R, Pomelli C, Ochterski JW, Martin RL, Morokuma K, Zakrzewski VG, Voth GA, Salvador P, Dannenberg JJ, Dapprich S, Daniels AD, Farkas Ö, Foresman JB, Ortiz JV, Cioslowski J, Fox DJ (2009). Gaussian 09, revision A.02.

[43] Becke AD (1993). J Chem Phys.

[44] Miehlich B, Savin A, Stoll H, Preuss H (1989). Chem Phys Lett.

[45] Lee C, Yang W, Parr G (1988). Phys Rev B.

[46] McLean AD, Chandler GS (1980). J Chem Phys.

[47] Krishnan R, Binkley JS, Seeger R, Pople JA (1980). J Chem Phys.

[48] Wachters AJH (1970). Chem Phys.

[49] Hay PJ (1977). J Chem Phys.

[50] Raghavachari K, Trucks GW (1989). J Chem Phys.

[51] Binning RC, Curtiss LA (1995). J Comput Chem.

[52] McGrath MP, Radom L (1991). J Chem Phys.

